# Effects of Composition Type and Activator on Fly Ash-Based Alkali Activated Materials

**DOI:** 10.3390/polym14010063

**Published:** 2021-12-24

**Authors:** Chan-Yi Lin, Tai-An Chen

**Affiliations:** Department of Harbor and River Engineering, National Taiwan Ocean University, Keelung City 202301, Taiwan; james980227@gmail.com

**Keywords:** alkali-activated materials, fly ash, GGBFs, alkali-equivalent content, amorphous, compressive strength, shrinkage

## Abstract

The compressive strengths of fly ash-based alkali-activated materials (AAM), produced using various activators of only sodium hydroxide, were measured. Fly ash-based AAM specimens, produced by mixing different kinds of fly ash and ground granulated blast-furnace slag (GGBFs) with an activator containing only sodium hydroxide, were cured at ambient temperature, and then placed in air for different numbers of days. The short- and long-term compressive strengths and shrinkage of fly ash-based AAM were measured and compared to one another. The effects of type of fly ash, alkali-equivalent content, GGBFs replace percentage, and ages on the compressive strengths and shrinkage of fly ash-based AAM were investigated. Even when different fly ash was used as the raw material for AAM, a similar compressive strength can be achieved by alkali-equivalent content, GGBFs replaces percentage. However, the performance of shrinkage due to different types of fly ash differed significantly.

## 1. Introduction

The latest UN figures suggest that despite current government commitments to reduce greenhouse gas emissions, atmospheric concentrations continue to rise, keeping the earth on a trajectory to levels of warming that will precipitate further environmental, social, and economic disruption and suffering on unprecedented scales [1]. Global GHG emissions increased by 1.5-fold since 1990. With several greenhouse gases in the atmosphere, including methane, nitrous oxide, and ozone-depleting substances, and greatly reducing CO_2_ is the most straightforward method to solve the continuous rise of air temperature [2]. As a major emitter of CO_2_ in the United States, the top three industries of CO_2_ emission are transportation, electricity, and manufacturing and building industry, which contribute 35%, 31%, and 16% of CO_2_ emissions, respectively, meaning the three industries account for more than 80% of total CO_2_ emissions [3].

Concrete is an extensively used construction material, and Portland cement is the foremost binder material, as well as the main source of concrete hydraulicity. However, a lot of CO_2_ is generated during the production process of any type of cement, which adversely affects the environment. Therefore, research is needed to find a substitute cement and reduce carbon emissions [4,5,6].

Geopolymer technology is a very effective industrial innovation, which uses the material made of aluminosilicate mineral and alkaline activity bath reaction, wherein most of the silicate mineral can be replaced by industrial waste. It was named “geopolymer” by Joseph Davidovits in 1979 [7]; the amorphous semicrystalline tri-dimensional alumino-silicates can rapidly form natural alumino-silicates solid materials under normal temperature through alkali reactivity. In addition, the geopolymer has an excellent heat resistance and fire resistance, and its carbon emission is lower than that of the conventional Portland cement [8]. Hence, it is regarded as a potential substitute building material that can be extensively used in industries, including construction in the future [9]. Many researchers also used alkali-activated materials (AAM) as a substitute for geopolymers. The difference between the chemical composition of geopolymers and AAMs is the calcium content, as calcium silicate hydrate does not become part of the polymerization product [10].

There are diverse raw materials of AAM, the industrial waste is usually used as raw material to save cost and reduce waste. The majority of the present solid wastes is the fly ash produced by coal-fired power plants, and the proportion of green power generation increases continuously; nevertheless, the coal-fired power generation still has marketability. Since the environmental pollution prevention strategies were established, people have used coal fly ash as a valuable material [11]. The fly ash can be transformed by an alkali-activated technique from waste into a construction material helpful to the environment [12]. The quality of coal fly ash varies with the composition and fineness of coal, fly ash collection form, and its storage method. Being the key to the quality of AAM, it is necessary to analyze the basic physical properties of each kind of fly ash.

The fly ash generally contains high amounts of Al-Si glass phases and a few crystal phases. With a high content of amorphous SiO_2_ and alumina, coal fly ash can react with alkaline solution rapidly [13], making it a suitable raw material of AAM.

The geopolymer is mainly composed of Si-O-Al structure, and its amorphous structure is different from synthetic zeolites. The geopolymerization mechanism involves the dissolution, migration, and polymerization of Si and Al precursors, and the addition of soluble silicate can accelerate the polymerization [14].

A major problem with alkali-activated materials is durability: if the degree of shrinkage is too severe, this may lead to cracks. Research findings indicated that alkali-activated high Ca substances usually have more severe drying shrinkage than cement matrix [15,16], and the physical properties of raw material are the key factor in the degree of dry shrinkage. This study employed three kinds of fly ash for a series of the same tests, aiming to distinguish heterogeneous fly ash.

## 2. Materials and Methods

### 2.1. Raw Materials

**Figure 1:**
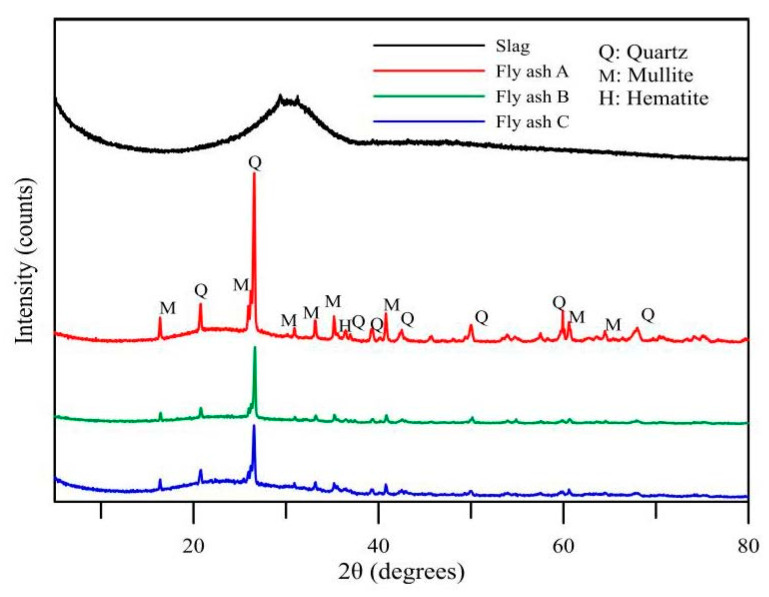
X-ray diffraction (XRD) patterns of fly ash and ground granulated blast-furnace slag.

**Figure 2:**
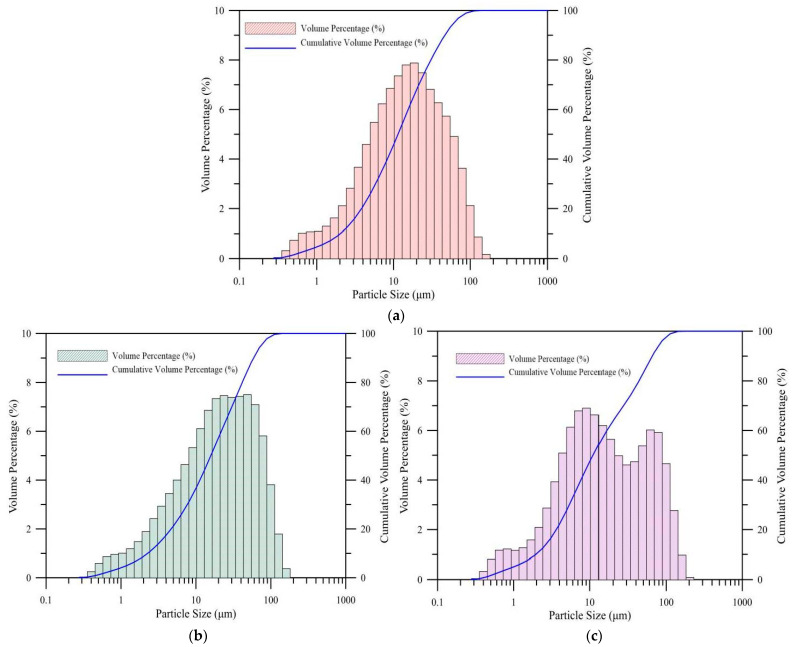
Particle size distribution of fly ash obtained from a laser diffraction particle size analyzer. (**a**) Fly ash A, (**b**) fly ash B, and (**c**) fly ash C.

In this study, we used three types of fly ash and their chemical properties are presented in Table 1. The X-ray diffraction (XRD) patterns of the powder shown in Figure 1, which were obtained at a scanning rate of 2θ/min and over a scanning range of 10°–80°, revealed different amorphous characteristics. The amorphous percentage of the four raw materials were 46.2, 56.7, 67.8, and 94.9%, respectively. The size distribution of the fly ash, as characterized using an INSITEC laser diffraction particle size analyzer, is shown in Figure 2. The mean geometric sizes of the three fly ash kinds were estimated to be 21.01 µm, 27.1 µm, and 26.5 µm, with standard deviations of 3.897, 3.568, and 5.758 respectively.

### 2.2. Activator

The fly ash and ground granulated blast-furnace slag (GGBFs) were alkali-activated by mixing with an activator in the production of AAM. The activator used here was a mixture of water and sodium hydroxide (NaOH) (reagent grade, 97% purity: Showa Chemical Industry Co., LTD, Tokyo, Japan). The chemical reaction, microstructure, and properties of an AAM can be affected dramatically by the amount of water in an activator. Therefore, the water/solid ratio was fixed at 0.3 for all fly ash-based AAM specimens. Only alkali-equivalent content parameters were employed in this study. The water/solid ratio (W/S) is the weight ratio of water to the sum of solid (includes powders and NaOH). The alkali-equivalent content, denoted by AE%, is defined as the weight fraction of Na_2_O to powders.

### 2.3. Sample Preparation, Mixing, and Curing

The activator plays an important role in determining the microstructure and properties of fly ash-based AAM specimens. To evaluate the effects of NaOH on the compressive strengths of fly ash-based AAM, activators with various alkali-equivalent content of AE% = 3%, 4%, 5%, 6%, 7%, and 8% were used in the production of fly ash-based AAM specimens, and replaced by 10%, 20% and 30% slag with a water/solid ratio of 0.3. The specimen proportions example is given in Table 2.

For each activator, the required amounts of NaOH, and water were weighed, mixed, and then placed in a container until room temperature was reached. Next, powders were added to the container and stirred vigorously for 2 min in a 5L Hobart mixer. After complete mixing, the AAM paste was poured into 3 × 3 × 3 cm steel molds, with a total of 18 cubes cast of each mix for the compressive strength tests and further compacted on a vibrating table (CONTROLS, frequency of 60 Hz) to get rid of any air bubbles. The steel molds were covered with plastic wrap to prevent the evaporation of moisture and then cured at an ambient temperature. The specimen size used for the shrinkage test was 25 × 25 × 285 mm, mixed, molded, and cured as same as above, with a total of three samples for each proportion. One day later, the specimens were demolded.

### 2.4. Pozzolanic Strength Activity Index

This test was carried out by reference to ASTM C311 [17], in which the 7- and 28-day compressive strengths of mortar cubes with a 20% mass replacement of cement by fly ash were compared to those of control without fly ash, at constant flow conditions. This was used to investigate the activity of different types of raw materials.

### 2.5. Workability

According to the mixing conditions in Section 2.3, the flow test was performed for the mixed specimen referring to ASTM C230 [18]. The paste was poured into the top split conical ring, and the flow table was bounced 25 times within 15 s after the conical ring was removed to measure the flowability of the mixture. The influence of the flowability of different slag substitution amounts was observed in different alkali-equivalent content conditions on workability.

### 2.6. Setting Time Test (Vicat Needle)

Firstly, in line with Section 2.3, AAM pasteis mixed by pouring in the conical ring (a height of 40 mm, an inside diameter at the bottom of 70 mm, and an inside diameter at the top of 60 mm). Periodic penetration tests are performed on this paste by allowing a 1 mm Vicat needle to settle into this paste. The Vicat initial time of setting is the time elapsed between the initial contact of cement and water and the time when the penetration is measured or calculated to be 25 mm. The Vicat’s final time of setting is the time elapsed between initial contact of cement and water and the time when the needle does not leave a complete circular impression in the paste surface. Since the setting time test is quite sensitive, the sample needed to be placed at a temperature of 23 °C and relative humidity of not less than 95%.

### 2.7. Compressive Strength Test

To determine the influence of the mixtures of different mix proportions on compressive strength, the specimens of all mix proportions were mixed according to Section 2.3 and made into a 3 × 3 × 3 cm specimen, which was hardened and cured under normal temperature. The compressive strength tests were carried out with reference to ASTM C109 [19]. The specimen was placed into the compression tester according to the curing ages of days 3, 7, 14, 28, 56, and 91 for compressive strength tests, and the strength was measured and recorded. The average of three specimens was used for each test.

### 2.8. Drying Shrinkage Test

The alkali-activated cementing material always has problems in volume stability, especially the alkali-activated slag, its dry shrinkage is quite large. Besides using fly ash as a base to produce AAM, this study also adopted a small amount of slag to replace fly ash. Therefore, it was necessary to measure the long-term volume stability. The drying shrinkage mold used in this study was a 25 × 25 × 285 mm steel die, and the measurement ages included days 3, 7, 14, 28, 56, and 91. The drying shrinkage test result showed that the volume stability of materials is very important for the usability of materials.

### 2.9. Microscopic Test

The raw materials were mixed according to Section 2.3. The reaction of fly ash in the specimen was considered more complete and the structure was more intact after 91 days of curing; hence, the specimen was cured at room temperature until 91 days as a microscopic test sample. The specimens made of three different materials were extracted from the mix proportion with the maximum compressive strength for microscopic test analysis. Firstly, the samples were pulverized and then vacuum dried until they reached a constant weight. Then, a portion of the sample was analyzed by XRD to determine the mineral components of the products. The XRD measurement was done with a D4 (Bruker) using a Co-Tube and equipped with a LynxEye detector. The settings were fixed divergence slits (0.5°), 0.04 rad Soller slits, and a step size of 0.02. The other part of the sample was placed on the Fourier transform infrared (FTIR) spectrometer for the recording of their infrared spectrum. The FTIR spectrum was recorded using a BRUKER, TENSOR II FT-IR Spectrometer over the wavelength range of 400 cm^−1^ to 4000 cm^−1^. The resolution of the measurement was 4 cm^−1^. After extracting and crushing some of the specimens, the fine particle samples were dried in a vacuum environment until they reached a constant weight. They were then placed in the Scanning Electron Microscope (SEM) to observe the extent of reaction of fly ash and the pore structure of products.

## 3. Results and Discussion

### 3.1. Pozzolanic Strength Activity Index

The test was performed referring to ASTM C311 [17], and the result is shown in Table 3. The activity indexes of the three kinds of fly ash exceeded 95% on Day 7, proving good activity, and the activity indexes were excellent at 117%, 125%, and 127% on Day 28. The activity index of GGBFS on Day 7 was 96%, and on Day 28 was 117%, meeting the Grade 120 furnace slag of ASTM C989 [20]. Data in Table 3 indicate that fly ash C has the highest activity index of all materials at 28 days and all three kinds of fly ash activity index are greater than GGBFs.

### 3.2. Flowability

**Figure 3:**
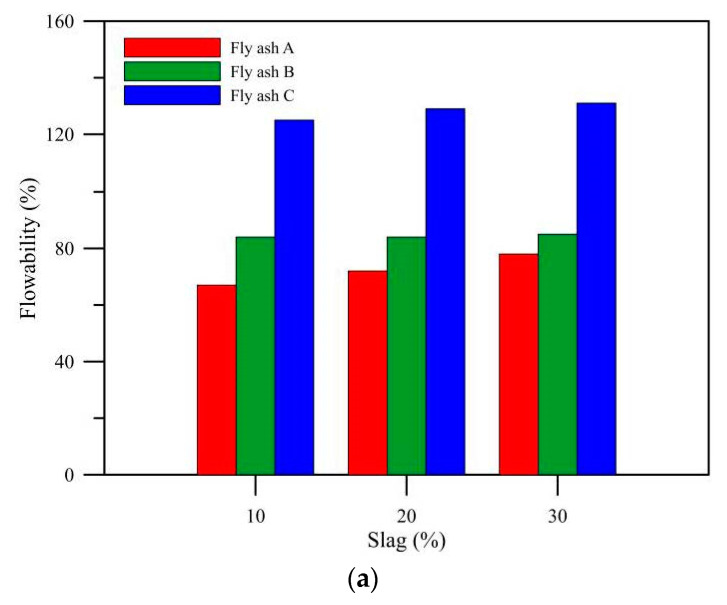

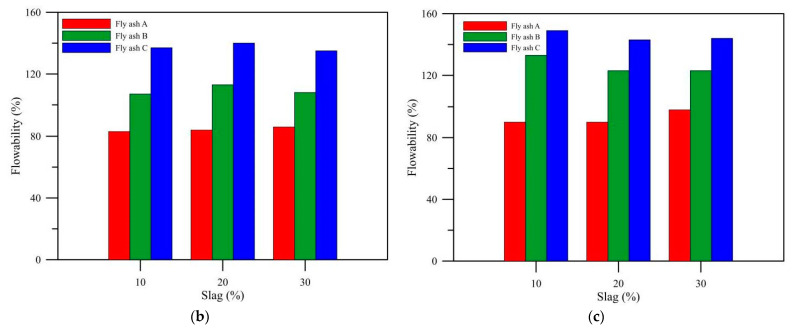
Flowability between different alkali-equivalents and different GGBFs replacements. (**a**) AE% = 3%; (**b**) AE% = 5%; (**c**) AE% = 8%.

The flow of mix proportions of AE% = 3, 5, and 8% of the three kinds of fly ash was tested as shown in Figure 3. As the setting time of fly ash C was much longer than that of fly ash A and B, it is less likely to cure and has a much higher flow rate than the other two kinds of fly ash. The flowability of various pastes increased with the alkali-equivalent content, because in the case of the same total mixing amount, the mixture with higher alkali-equivalent content has a lower total content of aggregate, and the flow of paste increased slightly with the slag substitution amount. However, when AE = 5 and 8%, and the slag replacement rate increased to 20%, the flow decreased because the rapid setting reaction of slag [21] reduced the flowability. However, when the slag replacement was 10%, the effect of slag on workability might be slighter than the effect of fly ash, as the fineness of the three kinds of fly ash was much lower than that of slag.

### 3.3. Setting Time

The setting time of mix proportions of AE% = 3, 5, and 8% of the three kinds of fly ash was tested. Wang et al. [21] reported a rapid setting problem of the slag. Consequently, our research modified the overlong setting time of pure fly ash.

**Figure 4:**
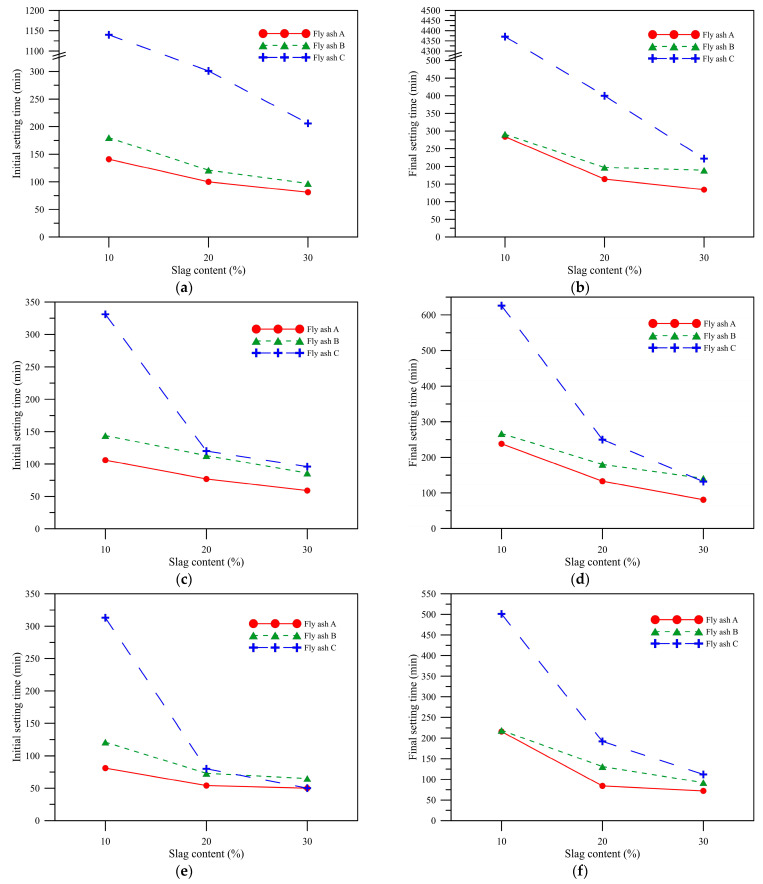
Initial and final setting times for different alkali equivalents and GGBFs replacements. (**a**) AE% = 3% initial setting times, (**b**) AE% = 3% final setting times, (**c**) AE% = 5% initial setting times, (**d**) AE% = 5% final setting times, (**e**) AE% = 8% initial setting times, (**f**) AE% = 8% final setting times.

As shown in Figure 4, the setting time of the AAM made of fly ash C was apparently longer than that of the other two kinds of fly ash, which results from different properties of the raw materials. Therefore, according to the XRF of the raw materials in Table 1, the CaO content in the fly ash C was 15.95%. This might not were caused by the insufficient CaO content to form AAM or other C-S-H colloids with silicates. Mortureux et al. [22] mentioned that the alkali-activated colloid in Ca form is likely to form in the case of high NaOH concentration; however, the colloid types formed by geopolymerization at a low alkali liquor concentration in this study were SiO_4_ and AlO_4_-tetrahedral structures. After QXRD by Rietveld quantitative analysis, the composition of the feed material is known, as shown in Section 2.1, the amorphous content of fly ash C was the highest at 67.8%, and that of fly ash B and fly ash A were 56.7% and 46.2%, respectively. This might be because the amorphous content was too high. In the case of low alkali liquor concentration and slag replacement rate, the originally unlikely polymerization was difficult to happen, and the paste could not be hardened. When AE% = 5 and 8%, as shown in Figure 4c–f, when the slag replacement rate was 20%, the mixture setting time could be shortened greatly. When the slag replacement rate was 30%, its effect on shortening the setting time was slighter.

According to the increasing alkali equivalent concentration, when AE% = 8%, as shown in Figure 4e,f, the overall setting time was shorter than that of AE% = 3%, corroborating the result of Gebreziabiher et al. [23], which indicated that the setting time can be shortened by using high concentration alkaline activator. When the alkali-equivalent content was increased to AE% = 8%, the paste of this study had the fastest polymerization, and the reaction rate increased with the slag replacement ratio. When AE% = 8% and the slag replacement rate was 30%, the initial setting time of the pastes made of fly ash A, fly ash B, and fly ash C was 50, 65, and 50 min, respectively; thus, there were few differences in the setting time of the three kinds of fly ash from different plants.

### 3.4. Compressive Strength

**Figure 5:**
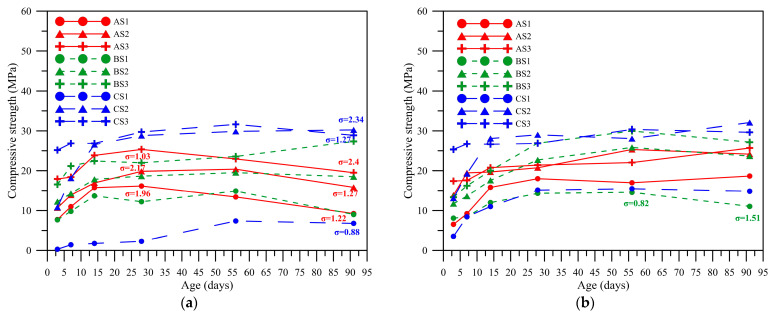

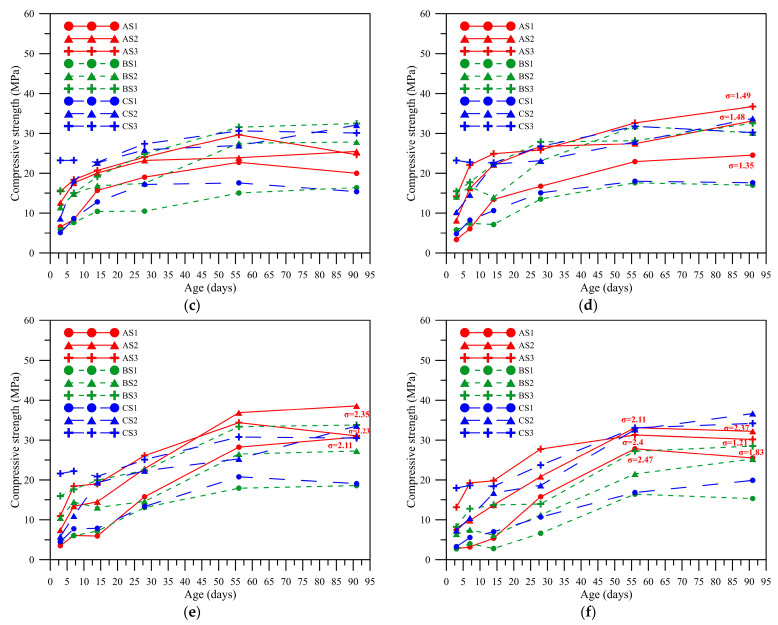
Compressive strength at different alkali equivalents for each proportion at different ages. (**a**) AE% = 3%, (**b**) AE% = 4%, (**c**) AE% = 5%, (**d**) AE% = 6%, (**e**) AE% = 7%, (**f**) AE% = 8%.

The compressive strength of all mixtures was tested. When AE% = 3%, as shown in Figure 5a, the compressive strengths of fly ash A were 16.15, 19.84, and 25.33 MPa with standard deviations of 1.96, 2.17, and 1.03 at the slag replacement rates of 10%, 20%, and 30% on Day 28. After Day 28, the compressive strength weakened as the age increased. The compressive strengths were 9.25, 15.82, and 19.51 MPa with standard deviations of 1.22, 1.27, and 2.4 on Day 91. The strength of fly ash B still increased when the slag replacement rate was 30% on Day 91, while the strength of replacement rate of 10% or 20% decreased slightly on Day 91. This is because the optimum amount of alkali required for fly ash differs from that required for slag, and there was no excess free alkali at 30% slag replacement to cause a late strength decline. The fly ash C had better compressive strength than the other two kinds when the slag replacement rates were 20% and 30%, with compressive strengths were 30.24 and 28.88 MPa, standard deviations of 2.34 and 1.27 on Day 91. However, when the slag replacement rate was 10%, the strength was much lower than that of the same mix proportion of the other two kinds of fly ash, with a compressive strength of 6.79 MPa and a standard deviation of 0.88. Based on the setting time of the mix proportions shown in Figure 4, it was suspected to be because the paste does not have adequate alkali content, resulting in inadequate reaction and polymerization and that the incompletely reacted alkali-activated liquid in the paste could not be retained. As a result, the strength did not yet improve after a longer age.

When AE% = 4%, as shown in Figure 5b, the strength development of various mix proportions became slow after Day 28. Except for the strength of fly ash B in the mix proportion of slag substitution amount of 10% decreased slightly during Day 56 to Day 91, with a compressive strength of 14.58 MPa at 56 days, a standard deviation of 0.82, and 11.05 MPa at 91 days, a standard deviation of 1.51. The other mix proportions did not have an obvious uptrend or downtrend. When AE = 5%, as shown in Figure 5c, the strength of various mix proportions was smooth after Day 56. Contrary to the smooth development in the strength when AE% = 4% on Day 28, the strength increased in the later stage with the increase in alkali-equivalent content.

When the alkali equivalent concentration was AE% = 6%, as shown in Figure 5d, the strength of various mix proportions increased with age. The strength of various mix proportions of fly ash A still increased significantly on Day 91, the compressive strengths of 24.53, 33.12, and 37.22 MPa with standard deviations of 1.35, 1.48 and 1.49 at the slag replacement rates of 10%, 20%, and 30% on Day 91. When AE% = 7 and 8%, as shown in Figure 5e,f, the optimum alkali-equivalent content of mix proportion of fly ash A was achieved. When AE% = 7% and the slag substitution amounts were 10%, 20%, and 30%, the strengths at 91 days were 30.75, 38.59, and 33.02 MPa, standard deviations of 2.11, 1.23 and 2.35, respectively, and increased gradually with age. When AE% = 8%, the strength began to decrease on Day 56. The compressive strengths were 27.84, 33.09, and 31.27 MPa with standard deviations of 2.47, 2.11, and 2.4 at the slag replacement rates of 10%, 20%, and 30% on Day 56, and 25.48, 32.19, and 30.17 MPa with standard deviations of 1.83, 2.37, and 1.21 on Day 91. The specimens of the mix proportions of fly ash B and fly ash C still had a good increase amplitude in the case of AE 8% from Day 56 to Day 91, and the strength could be increased by increasing the alkali equivalent concentration.

### 3.5. Drying Shrinkage

**Figure 6:**
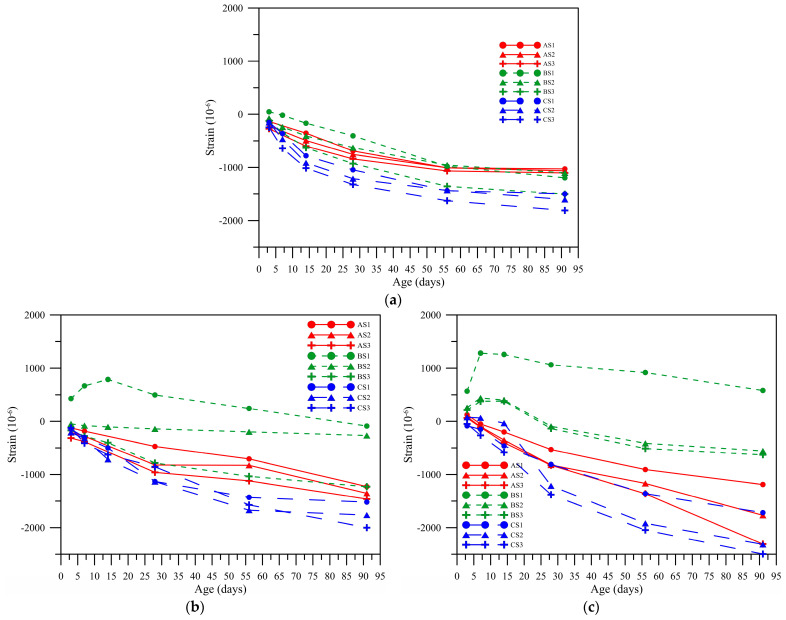
Drying and shrinkage of various proportions with age at different alkali equivalents. (**a**) AE% = 3%, (**b**) AE% = 5%, (**c**) AE% = 8%.

This study employed AE% = 3, 5, and 8% and the alkali-equivalent content in Figure 6 for the drying shrinkage test. When AE% = 5%, as shown in Figure 6b, and the slag replacement rate was 10%, the fly ash B expanded acutely and shrank gradually after the age of 14 days. In the mix proportions of fly ashes A and C, the shrinkage amplitude increased gradually with age; the shrinkage amplitude was larger than that of AE 3%. When AE% = 8% as shown in Figure 6c, and the slag replacement rate of the fly ash B was 10%, the fly ash B exhibited more severe expansion than AE 5%, and the expansivity was 0.5813% at the age of 91 days.

The mix proportion of fly ash C had a severe shrinkage. At the age of 91 days, for AE% = 3% as shown in Figure 6a, when the slag substitution amounts were 10, 20, and 30%, the amounts of change in length were −1.718%, −2.315%, and −2.497%, respectively.

The shrinkage of mix proportion of fly ash A was next to fly ash C, when AE% = 3% and the slag substitution amounts were 10, 20, and 30%, the amounts of change in length are −1.1893%, −1.768%, and −2.309%, respectively.

According to the test result, the higher the GGBFs replacement rate was, the more severe the shrinkage, which is due to the very large degree of autogenous shrinkage of the stone itself [24]. The higher the alkali-equivalent content of fly ash C and fly ash A was, the larger the amount of deformation from shrinkage was. However, the expansion of fly ash B increased with alkali-equivalent content. According to the compound composition of various raw materials in Table 1, the highest CaO content was in fly ash C at 16.88%, so its expansion may be the volume expansion resulting from the reaction of a high content of F-CaO and water.

### 3.6. X-ray Diffraction Analysis

**Figure 7:**
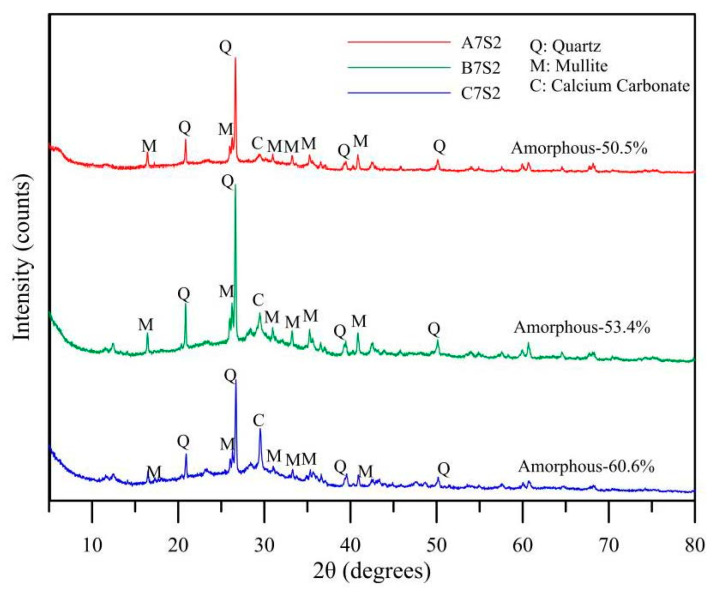
XRD analysis of different fly ash mixtures at alkali equivalent = 7% and 20% GGBFs replacement.

The maximum compressive strength of the specimens in this study occurred in the fly ash A specimen when AE% = 7% and slag replacement rate was 20%; hence, the specimens of three kinds of fly ash and the same mix proportion were extracted for XRD analysis. Figure 7 shows the diffractograms of three results. When 2θ = 25°–26°, there was an obvious quartz mineral diffraction peak, forming the mineral components of the main crystal with Mullite. When 2θ = 28°, the presence of calcium carbonate was detected, meaning that Ca in the raw material had not fully entered into polymerization to generate strong minerals.

### 3.7. Scanning Electron Microscopy Observation

**Figure 8:**
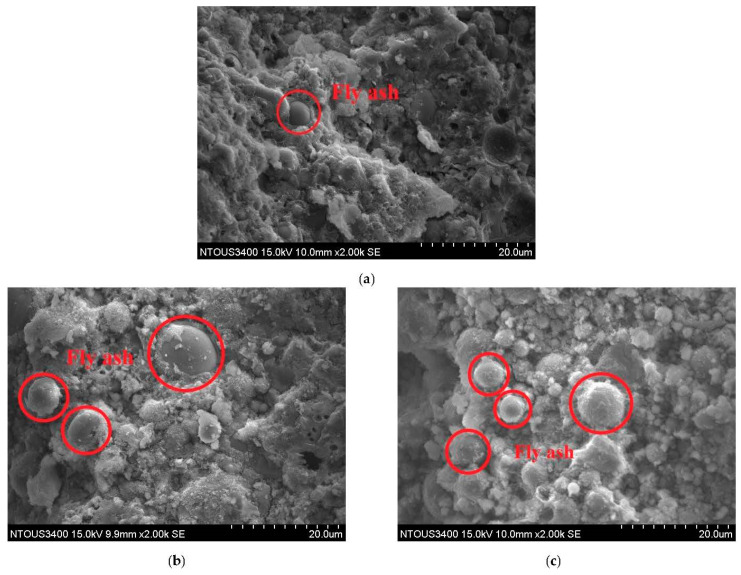
Scanning Electron Microscope of different fly ash mixtures at alkali equivalent = 7% and 20% GGBFs replacement. (**a**) Fly ash A, (**b**) fly ash B, and (**c**) fly ash C.

The mixture of three different materials in the same mix proportion was cured under ordinary temperature for 91 days before SEM tests. As shown in Figure 8, the fly ash A sample exhibited fewer residual fly ash particles than fly ash B and fly ash C, and its texture was denser. This result is the same as the result of compressive strength in Figure 5. The fly ash A exhibited the highest compressive strength. As fly ash C contained the most unreacted fly ash, the alkali-activated liquid was suspected to increase continuously, the more spherical vitreous surface of fly ash could be damaged to dissolve the internal rich silica constituent, and a higher strength specimen could possibly be polymerized. Subsequently, different kinds of fly ash possessed different optimum alkali-equivalent contents, and the reactive fly ash contents in the same mix proportion were different.

### 3.8. FTIR Analysis

**Figure 9:**
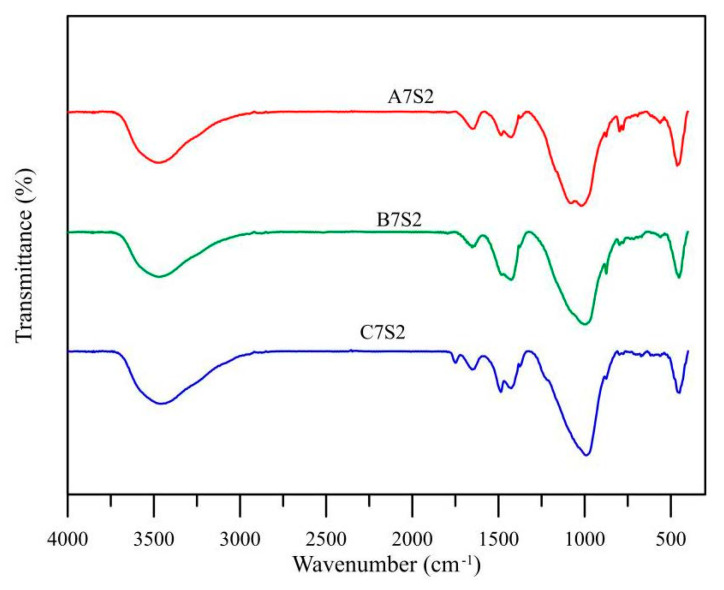
Fourier Transform Infrared Spectroscopy analysis of different fly ash mixtures at alkali equivalent = 7% and 20% GGBFs replacement.

Figure 9 shows the infrared spectrogram of the three specimens. An apparent peak was observed at 1020 cm^−1^, which represents the Si-O and Si-O-Si stretching vibration absorption peak [25]. There is a micro peak at 875 cm^−1^, wherein the absorption band and 1400 cm^−1^ are recognized as the vibration of the O-C-O bond of carbonate [26]. As the raw material was free of carbonate, certain carbonization might have occurred during the polymerization process. This result matched with the carbonate product in the XRD analysis shown in Figure 7, it is universally regarded as the cause for deterioration of the geopolymerization product [27].

## 4. Conclusions

(1) The ultimate strength of specimens occurred in the specimen of fly ash A in this study. The activity index on Day 28 of fly ash A was only 117%, and the amorphous form was the lowest among the three raw materials, indicating the activity index and amorphous content are not in absolute relation to AAM strength.
(2) The optimum alkali-equivalent content in fly ash A was AE% = 7%, with the best compressive strength 38.58 MPa at 20% slag substitution amount. The mix proportion with the second-best compressive strength occurred in fly ash C, at AE% = 8%. The compressive strength was 36.63 MPa when the slag substitution amount was 20%. The fly ash B exhibited the lowest strength. The compressive strength was 33.77 MPa when AE% = 7% and the slag substitution amount was 30%.
(3) The fly ash C had the longest setting time and was the most amorphous. Without adequate alkali-equivalent content and slag substitution amount, the polymerization was harder to happen.
(4) According to the drying shrinkage test, the AAM of fly ash C had very large shrinkage because it was unlikely to set. The AAM of fly ash B exhibited too high f-CaO content, as it reacts with water and expands. Therefore, the fly ash material greatly influences the basic properties.
(5) The mixtures made of heterogeneous fly ash have their optimal mix proportions, with appropriate alkali-equivalent content and slag substitution amount, any kind of fly ash can form higher strength AAM specimens.

## Figures and Tables

**Table 1:** Chemical properties of fly ash produced and ground granulated blast-furnace slag (GGBFs) in this study.

										Unit: %
Material	SiO_2_	Fe_2_O_3_	Al_2_O_3_	CaO	MgO	TiO_2_	K_2_O	SrO	SO_3_	LOI
Fly ash A	49.89	24.71	7.01	6.31	-	4.97	2.42	0.92	0.43	1.6
Fly ash B	41.68	19.56	10.09	16.88	-	5.05	2.81	1.54	0.52	1.87
Fly ash C	35.53	30.5	4.89	15.95	-	4.97	3.43	1.72	1.07	4.89
GGBFs	33.53	0.27	14.85	40.53	7.17	-	-	-	-	0.08

**Table 2:** Example of proportion at AE% = 8%.

						Unit: g
Mix Designation	Water	NaOH	Fly Ash A	Fly Ash B	Fly Ash C	GGBFs
AS1	165.5	51.6	450	-	-	50
AS2	165.5	51.6	400	-	-	100
AS3	165.5	51.6	350	-	-	150
BS1	165.5	51.6	-	450	-	50
BS2	165.5	51.6	-	400	-	100
BS3	165.5	51.6	-	350	-	150
CS1	165.5	51.6	-	-	450	50
CS2	165.5	51.6	-	-	400	100
CS3	165.5	51.6	-	-	350	150

**Table 3:** Pozzolanic strength activity index and Blaine Specific surface area for each material.

Pozzolanic Strength Activity Index	7 Days	28 Days	Blaine Specific Surface (m^2^/kg)
Fly ash A	104%	118%	417
Fly ash B	104%	125%	357
Fly ash C	95%	127%	627
GGBFs	96%	117%	580

## References

[1] Special Report: Global Warming of 1.5 °C. https://www.ipcc.ch/sr15.

[2] Hoegh-Guldberg O., Jacob D., Taylor M., Bolaños T.G., Bindi M., Brown S., Camilloni I.A., Diedhiou A., Djalante R., Ebi K. (2019). The human imperative of stabilizing global climate change at 1.5 C. Science.

[3] Overview of Greenhouse Gases. https://www.epa.gov/ghgemissions/overview-greenhouse-gases#carbon-dioxide.

[4] Dueramae S., Tangchirapat W., Chindaprasirt P., Jaturapitakkul C., Sukontasukkul P. (2020). Autogenous and drying shrinkages of mortars and pore structure of pastes made with activated binder of calcium carbide residue and fly ash. Constr. Build. Mater..

[5] Namarak C., Tangchirapat W., Jaturapitakkul C. (2018). Bar-concrete bond in mixes containing calcium carbide residue, fly ash and recycled concrete aggregate. Cem. Concr. Compos..

[6] Gholampour A., Ho V.D., Ozbakkaloglu T. (2019). Ambient-cured geopolymer mortars prepared with waste-based sands: Mechanical and durability-related properties and microstructure. Compos. Part B Eng..

[7] Davidovits J. (2002). Years of successes and failures in geopolymer applications. Market trends and potential breakthroughs. Proceedings of the Geopolymer 2002 Conference.

[8] Deb P.S., Nath P., Sarker P.K. (2014). The effects of ground granulated blast-furnace slag blending with fly ash and activator content on the workability and strength properties of geopolymer concrete cured at ambient temperature. Mater. Des..

[9] Giannopoulou I., Dimas D., Maragkos I., Panias D. (2009). Utilization of metallurgical solid by-products for the development of inorganic polymeric construction materials. Glob. NEST J..

[10] Mehta A., Siddique R., Ozbakkaloglu T., Shaikh F.U.A., Belarbi R. (2020). Fly ash and ground granulated blast furnace slag-based alkali-activated concrete: Mechanical, transport and microstructural properties. Constr. Build. Mater..

[11] Álvarez-Ayuso E., Querol X., Plana F., Alastuey A., Moreno N., Izquierdo M., Font O., Moreno T., Diez S., Vázquez E. (2008). Environmental, physical and structural characterisation of geopolymer matrixes synthesised from coal (co-) combustion fly ashes. J. Hazard. Mater..

[12] Diaz E.I., Allouche E.N., Eklund S. (2010). Factors affecting the suitability of fly ash as source material for geopolymers. Fuel.

[13] Rattanasak U., Chindaprasirt P. (2009). Influence of NaOH solution on the synthesis of fly ash geopolymer. Miner. Eng..

[14] Pereira C.F., Luna Y., Querol X., Antenucci D., Vale J. (2009). Waste stabilization/solidification of an electric arc furnace dust using fly ash-based geopolymers. Fuel.

[15] Collins F., Sanjayan J.G. (2000). Effect of pore size distribution on drying shrinking of alkali-activated slag concrete. Cem. Concr. Res..

[16] Neto A.A.M., Cincotto M.A., Repette W. (2008). Drying and autogenous shrinkage of pastes and mortars with activated slag cement. Cem. Concr. Res..

[17] (2018). Standard Test Methods for Sampling and Testing Fly Ash or Natural Pozzolans for Use in Portland-Cement Concrete.

[18] (2021). Standard Specification for Flow Table for Use in Tests of Hydraulic Cement.

[19] (2021). Standard Test Method for Compressive Strength of Hydraulic Cement Mortars (Using 2-in. or [50-mm] Cube Specimens).

[20] (2018). Standard Specification for Slag Cement for Use in Concrete and Mortars.

[21] Wang S.D., Pu X.C., Scrivener K.L., Pratt P.L. (1995). Alkali-activated slag cement and concrete: A review of properties and problems. Adv. Cem. Res..

[22] Mortureux B., Hornain H., Gautier E., Regourd M. (1980). Comparaison de la réactivité de différentes pouzzolanes. Proceedings of the 7th International Conference on the Chemistry of Cement.

[23] Gebregziabiher B.S., Thomas R.J., Peethamparan S. (2016). Temperature and activator effect on early-age reaction kinetics of alkali-activated slag binders. Constr. Build. Mater..

[24] Hojati M., Radlińska A. (2017). Shrinkage and strength development of alkali-activated fly ash-slag binary cements. Constr. Build. Mater..

[25] Hajimohammadi A., Provis J.L., van Deventer J.S. (2011). Time-resolved and spatially-resolved infrared spectroscopic observation of seeded nucleation controlling geopolymer gel formation. J. Colloid Interface Sci..

[26] Bernal S.A., Provis J.L., Rose V., de Gutierrez R.M. (2011). Evolution of binder structure in sodium silicate-activated slag-metakaolin blends. Cem. Concr. Compos..

[27] Chen T.A. (2020). Optimum curing temperature and duration of alkali-activated glass inorganic binders. J. Chin. Inst. Eng..

